# Supplementary material for: Atomic-scale age resolution of planetary events
Source: Nat Commun. 2017 May 26;8:15597. doi: 10.1038/ncomms15597 (PMC5477514; doi:10.1038/ncomms15597)
Supplement: Supplementary Information — Supplementary Figures and Supplementary Tables [file ncomms15597-s1.pdf]

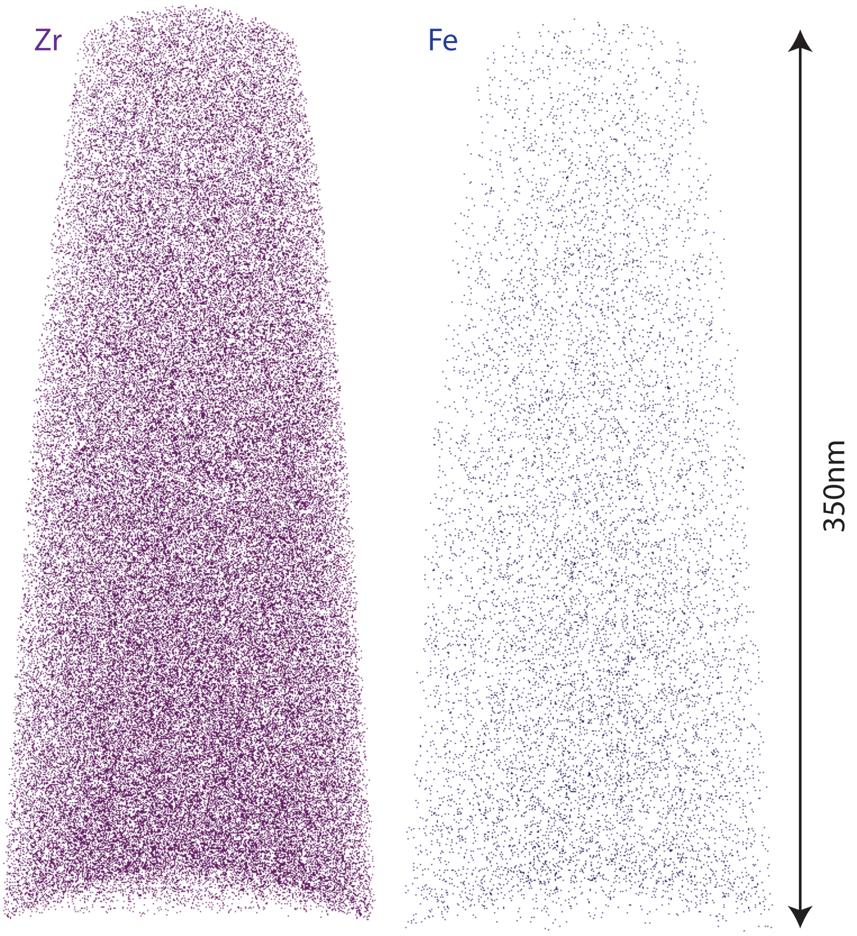

Supplementary Figure 1: Three-dimensional reconstruction a single microtip specimen of the Phalaborwa baddeleyite standard. APT reconstruction of a single tip, displaying homogenous distribution of Fe and Zr atoms throughout the specimen. Models display no vertical exaggeration.

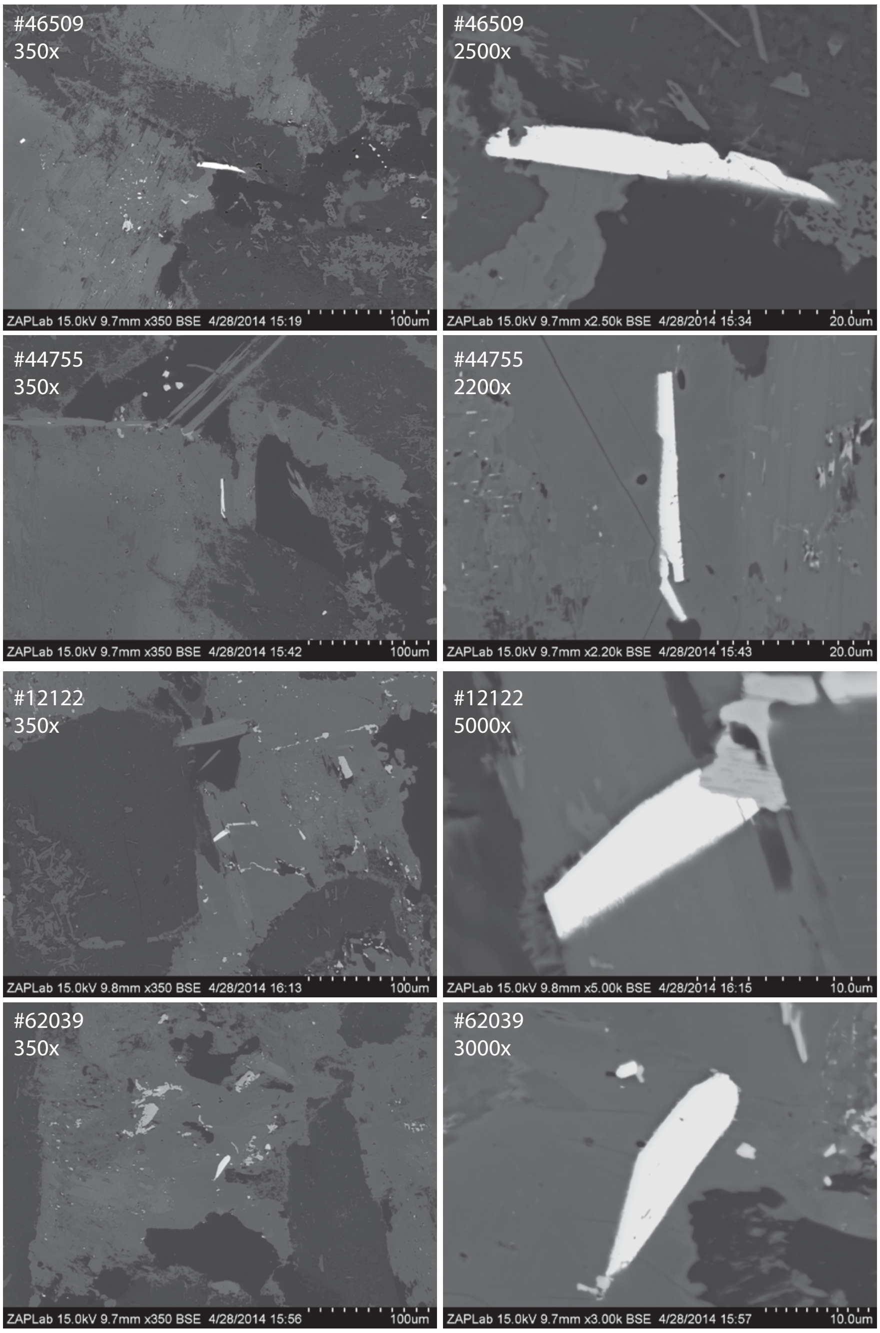

Supplementary Figure 2: Back-scatter electron images of four baddeleyite grains from highly shocked Matachewan sample JD12SUD14. Low (350x) and high (2200 – 5000x) magnification BSE images of baddeleyite grains #46509, #44755, #12122 and #62039. For this study, eleven microtip specimen have been produced from grains #46059 and #44755 for analysis by atom probe tomography.

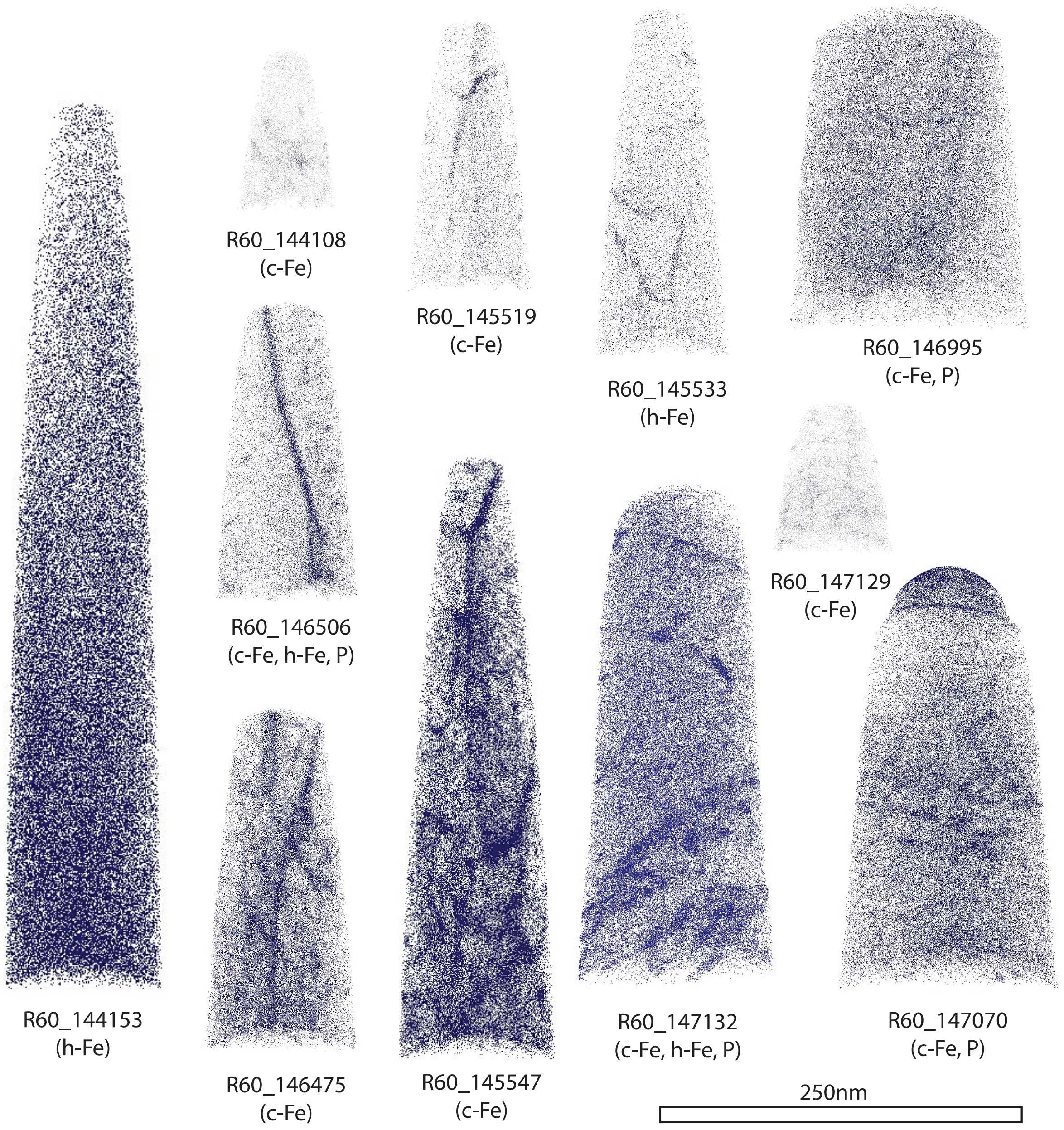

Supplementary Figure 3: Three-dimensional reconstructions of all eleven atom probe microtip specimen incorporated into this study. All images highlight Fe distribution within the dataset and are scaled to match one another. Models display no vertical exaggeration. Each specimen is labeled with its associated microtip number and shorthand notation as to its constituent nanostructural domains. (c-Fe = clustered-Fe; h-Fe = homogenous-Fe; P = planar). In total, the eleven microtip specimen yield eight homogenous-Fe domains, four clustered-Fe domains, and four planar or curviplanar features.

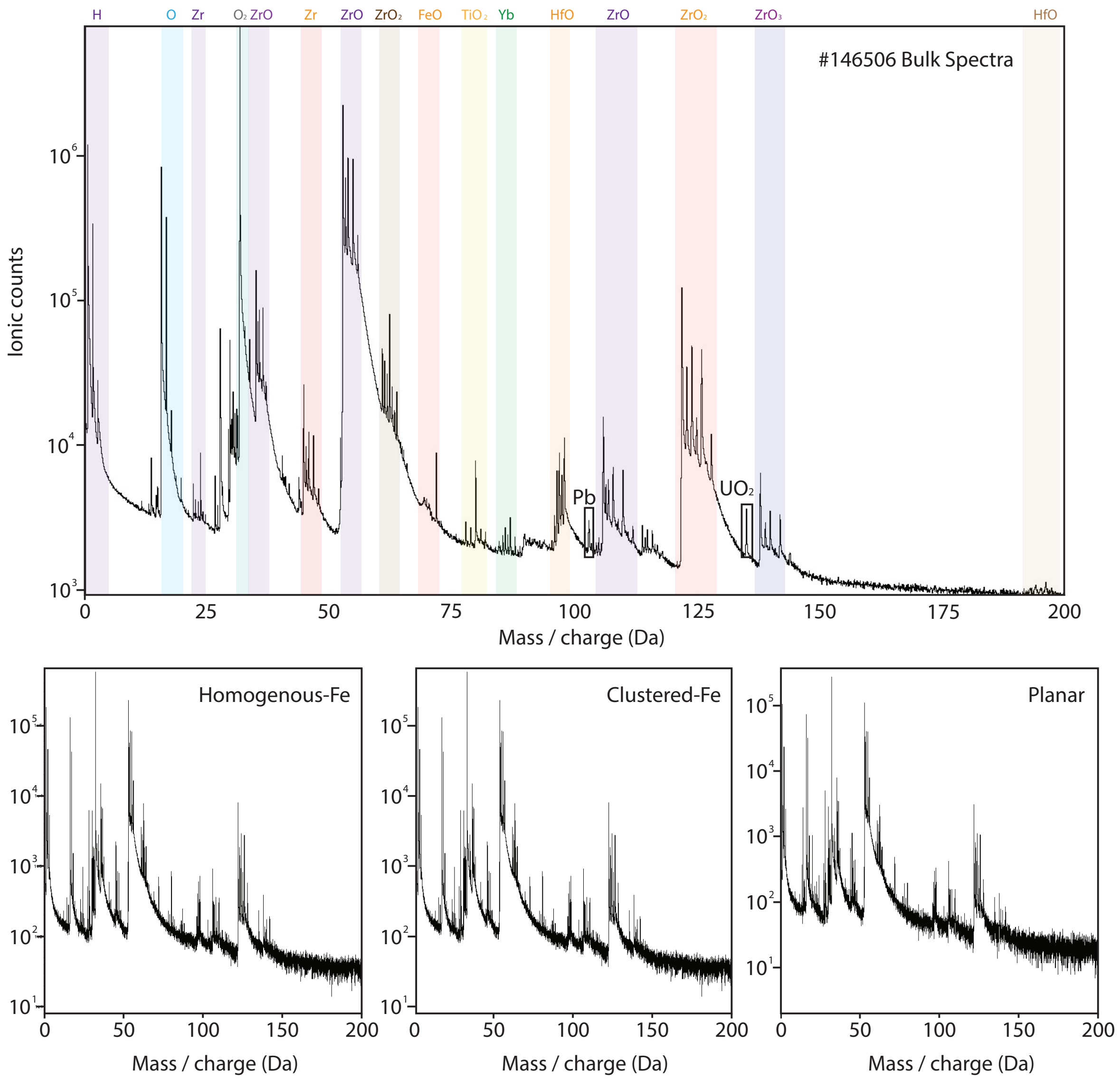

Supplementary Figure 4: Labelled mass-to-charge spectra for bulk microtip #146506, with smaller scale images of spectra generated from homogenous-Fe, clustered-Fe and planar domains. Major families of species are colored and labelled for reference, while the predominate peaks for ^{206}Pb (103 Da) and ^{238}U (135 Da) are highlighted for reference. All spectra are visualized using 0.2nm binning intervals, while all background and signal are displayed up to 200 Da.

Microtip	^{206}Pb	^{238}U	^{206}Pb/^{238}U	2σ	^{206}Pb/^{238}U Age (Ma)	2σ
144153	1776	3870	0.4589	0.2026	2435	1075
147132	2810	6499	0.4324	0.1008	2316	540
145533	665	1547	0.4297	0.1697	2304	910
145519	269	737	0.3647	0.2498	2004	1373
147070	2484	7047	0.3526	0.0837	1947	462
146506	1483	4246	0.3493	0.0741	1931	409
145547	3221	9544	0.3375	0.0425	1874	236
146475	3076	9296	0.3309	0.0405	1843	225
144108	498	1554	0.3204	0.0856	1792	478
146995	4870	15464	0.3149	0.0514	1765	288
147129	950	3666	0.2591	0.0545	1485	313

Supplementary Table 1: Bulk ^{206}Pb and ^{238}U data from eleven microtip specimens extracted from two baddeleyite grains (#44755 and #46059) of the Matachewan dyke swarm, Ontario, Canada. All counts represent background-corrected, ranged ionic counts of ^{206}Pb and ^{238}U within the bulk microtip samples. All reported errors (2σ) have been propagated through from raw and background counting statistics as outlined in Methods.

Clustered-Fe	^{206}Pb	^{238}U	^{206}Pb/^{238}U	2σ	^{206}Pb/^{238}U Age (Ma)	2σ
146506 (SD)	491	1394	0.3525	0.1154	1947	637
146475 (WT)	3076	9296	0.3309	0.0405	1843	225
145547 (WT)	3221	9544	0.3375	0.0425	1874	236
146995 (SD)	1221	3532	0.3456	0.1047	1914	580
144108 (WT)	498	1554	0.3204	0.0856	1792	478
145519 (SD)	151	485	0.3112	0.2413	1747	1354
147129 (SD)	457	1361	0.3355	0.1064	1865	591
147070 (SD)	2113	6427	0.3287	0.0789	1832	440
Homogenous-Fe						
146506 (SD)	365	764	0.4776	0.3015	2517	1589
145533 (WT)	665	1547	0.4297	0.1697	2304	910
147132 (SD)	464	985	0.4712	0.2444	2489	1291
144153 (WT)	1776	3870	0.4589	0.2026	2435	1075
Planar						
146506 (SD)	452	1768	0.2555	0.0712	1467	409
147132 (SD)	212	844	0.2514	0.1194	1445	687
146995 (SD)	2090	8628	0.2422	0.0579	1398	334
147070 (SD)	159	688	0.2309	0.2454	1339	1424

Supplementary Table 2: Nanostructurally subdivided ^{206}Pb and ^{238}U data from all eleven microtip specimens. Specimen numbers are followed by shorthand notation as to whether they represent a whole tip (WT) or subdomain (SD) dataset. All reported errors (2σ) have been propagated through from raw and background counting statistics as outlined in Methods.
